# Evaluating Potential Therapeutic Targets and Drug Repurposing Based on the Esophageal Cancer Subtypes

**DOI:** 10.3390/ph18081181

**Published:** 2025-08-11

**Authors:** Jongchan Oh, Jongwon Han, Heeyoung Lee

**Affiliations:** 1 Department of Pharmacy, Inje University, Gimhae 50843, Gyeongnam, Republic of Korea; whdcks4698@naver.com (J.O.); hanko123@naver.com (J.H.); 2 Inje Institute of Pharmaceutical Sciences and Research, Inje University, Gimhae 50843, Gyeongnam, Republic of Korea

**Keywords:** esophageal cancer, esophageal adenocarcinoma, esophageal squamous cell carcinoma, biomarkers, hub genes, drug repurposing, MEK inhibitors

## Abstract

**Background:** Esophageal cancer (EC), including esophageal adenocarcinoma (EAC) and esophageal squamous cell carcinoma (ESCC), remains a lethal malignancy with limited molecularly tailored treatment options. Due to substantial histologic and transcriptomic differences between subtypes, therapeutic responses often vary, underscoring the need for subtype-stratified analysis and precision drug discovery. **Methods:** We integrated transcriptomic data from GEO and TCGA to identify differentially expressed genes (DEGs) specific to EAC, ESCC, and their shared profiles. Functional enrichment (GO, KEGG) and protein–protein interaction (PPI) network analyses were conducted to extract hub genes using DAVID, STRING, and Cytoscape. Survival associations were evaluated using TCGA-ESCA and UALCAN. Drug repurposing was performed using L1000FWD, L1000CDS2, and SigCom LINCS. **Results:** We identified 79, 59, and 17 hub genes in the DEG-EAC, DEG-ESCC, and DEG-EAC&ESCC datasets, respectively. In EAC, 16 novel hub genes including SCARB1, SERPINH1, and DSC2 were discovered, which had not been previously implicated in this subtype. These genes were significantly enriched in pathways related to extracellular matrix (ECM) remodeling and epithelial structure. In addition, shared hub genes across EAC and ESCC—such as COL1A1, SPARC, and MMP1—were enriched in ECM organization and cell adhesion processes, highlighting convergent tumor–stroma interactions. Drug repositioning analysis consistently prioritized MEK inhibitors, trametinib and selumetinib, as potential therapeutic candidates across all DEG datasets. **Conclusions:** This study presents a comprehensive, subtype-stratified transcriptomic framework for EC, identifying both unique and shared hub genes with potential functional relevance to ECM dynamics. Our findings suggest that ECM remodelers may serve as therapeutic targets, and highlight MEK inhibition as a promising, yet exploratory, repurposing strategy. While these results offer a molecular foundation for future precision oncology efforts in EC, further validation through proteomic analysis, functional studies, and clinical evaluation is warranted.

## 1. Introduction

Esophageal cancer (EC) is often diagnosed at an advanced stage and shows high recurrence and poor clinical outcome with low survival rate; it is known for its rapid progression [1]. Integrating various factors such as race, region, and environmental issues have led to limited treatment options for EC patients in early stages [1,2]. Given the importance of early detection and intervention, it is essential to move beyond empirical approaches that treat all patients as if they have the same disease behavior in EC patients [1,3,4]. Furthermore, in previous studies, the complex histology and pathology of esophageal cancer (EC) subtypes, esophageal adenocarcinoma (EAC) and esophageal squamous cell carcinoma (ESCC), led to inconsistent therapeutic responses, resulting in varied outcomes in EC patient care [1]. According to Xi et al., among patients without a pathologic complete response, locoregional recurrences were more frequent in ESCC than in EAC (16.7% vs. 6.3%; *p* < 0.001), whereas distant recurrences were more common in EAC than in ESCC (32.5% vs. 17.5%; *p* = 0.002), but no significant difference in overall survival (OS) was observed between the two subtypes (*p* = 0.772) [5]. A CROSS trial showed overall survival with neoadjuvant chemoradiotherapy was greater than ESCCs (46%, 95% confidence interval [CI] 33–64) than EAC (36%, 95% CI 29–45) [6]. Considering these inconsistent therapeutic responses and histologic differences, increasing attention has been directed toward identifying distinct molecular signatures, including gene expression patterns [7,8]. Li et al. comprehensively compared the transcriptomic landscapes of ESCC and EAC at multiple levels and demonstrated that differential patterns of post-transcriptional events, such as alternative splicing and alternative polyadenylation, play crucial roles in shaping the tumor microenvironment in these cancers, although they did not show any gene expression differences [7]. In addition, Wang et al. identified similarities and differences in gene expression between EAC and ESCC, reporting 10 hub genes with subtype-specific expression patterns based on the analysis of a single dataset, GSE26886 [8]. However, the study was limited by sample size, which precluded more comprehensive subtype-specific conclusions. Similarly, King et al. examined metabolic and immunologic differences between the two subtypes, but the small sample size and the narrow focus on specific histological pathways may have limited the identification of a broader set of subtype-specific key genes [9]. These prior inconsistencies highlight that EAC and ESCC may represent biologically shared and distinct diseases. To resolve current uncertainties in treatment strategies, a deeper understanding of the molecular underpinnings of both subtypes is essential for more rational and effective management of esophageal cancer.

Through improving molecular understanding, the identification of hub genes—genes with high connectivity in protein–protein interaction (PPI) networks—has emerged as a promising strategy for uncovering critical molecular drivers of cancer progression [10,11]. These hub genes not only provide insights into tumor biology but also serve as potential therapeutic targets [12]. To advance the development of subtype-specific treatments for esophageal cancer (EAC and ESCC), many studies have employed drug repositioning, repurposing existing drugs to target cancer-specific hub genes [13,14,15]. However, these studies did not employ integrating subtype-specific and shared datasets to analyze comprehensive drug screening platforms in esophageal cancer.

Thus, to our knowledge, this is the first study to identify subtype-specific and shared hub genes in EAC and ESCC by leveraging a larger and more extensive collection of transcriptomic datasets, and concurrently to explore potential therapeutic agents through drug repositioning strategies using computational drug–gene interaction platforms.

## 2. Results

**Figure 1:**
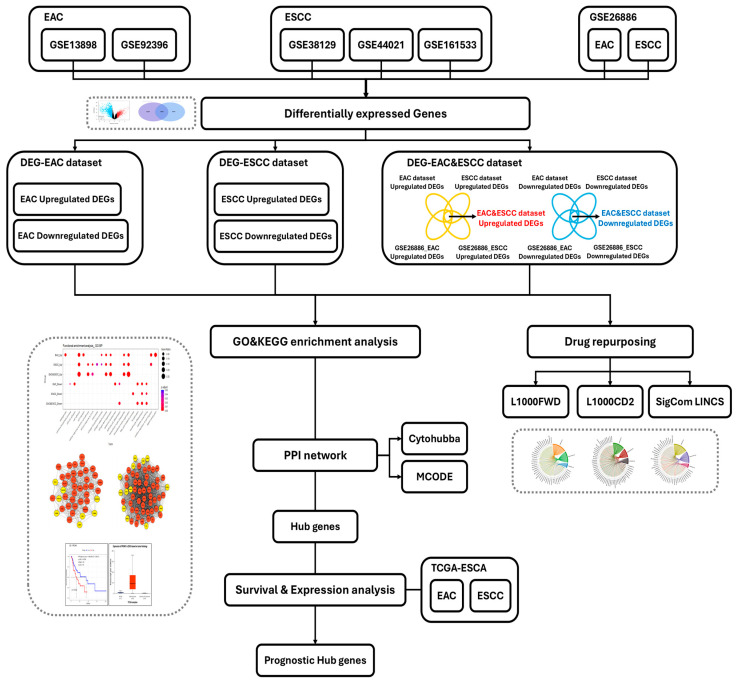
Schematic overview of the study workflow.

The schematic overview of the study workflow is illustrated in Figure 1.

### 2.1. Identification of Common DEGs in EAC and/or ESCC Datasets

**Figure 2:**
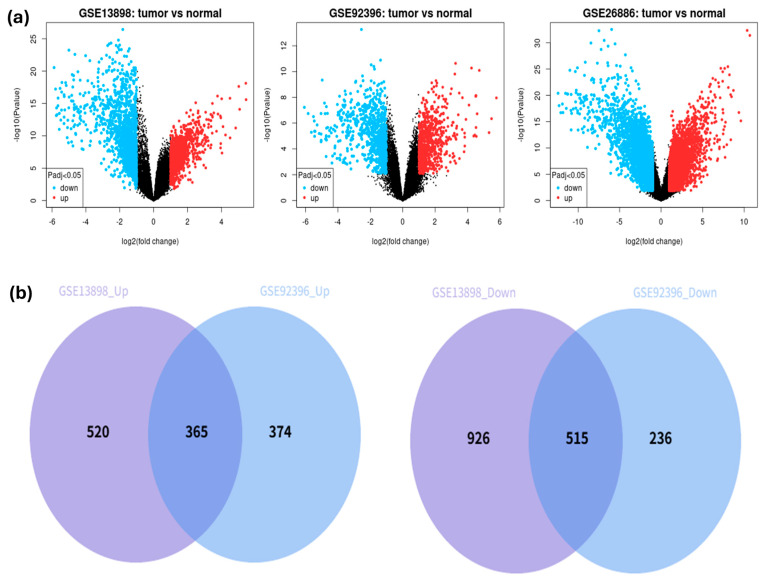
Identification of DEGs: (**a**) Volcano plots for comparing EAC vs. normal samples; (**b**) Venn diagrams showing overlapping upregulated and downregulated DEGs within the EAC datasets.

To identify differentially expressed genes (DEGs) specific to EAC and/or ESCC, we analyzed six publicly available GEO datasets. Two datasets—GSE13898 and GSE92396—represented EAC and included a total of 76 tumor and 37 normal samples. Three datasets—GSE38129, GSE44021, and GSE161533—represented ESCC and consisted of 131 tumor and 131 normal samples. In addition, GSE26886 contained gene expression profiles for both EAC (21 samples) and ESCC (9 samples), along with 19 normal samples. Volcano plots illustrating differential gene expression in each dataset were generated using GEO2R for EAC (Figure 2a) and ESCC (Supplementary Figure S1). Venn diagram analysis revealed 880 consistently dysregulated DEGs in the DEG-EAC dataset (365 upregulated, 515 downregulated) (Figure 2b) and 599 in the DEG-ESCC dataset (300 up, 299 down) (Supplementary Figure S2a). Separately, GSE26886 yielded 1631 upregulated and 1851 downregulated genes shared between EAC and ESCC samples. From our analysis, 64 upregulated and 138 downregulated DEGs were shared between the DEG-EAC and DEG-ESCC datasets. To further confirm these shared DEGs by intersecting with the GSE26886 dataset, a total of 188 common DEGs (52 upregulated, 136 downregulated) were identified across both EAC and ESCC samples (Supplementary Figure S2b).

### 2.2. GO and KEGG Pathway Enrichment Analysis of Common DEGs from EAC, ESCC, and EAC&ESCC Datasets

**Figure 3:**
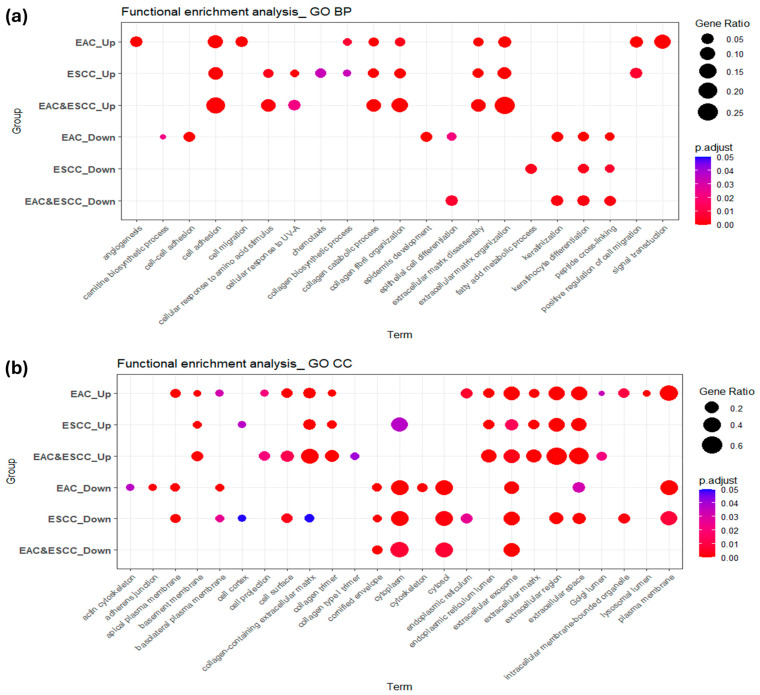
Enrichment patterns of DEGs after functional enrichment analysis based on EAC, ESCC, and EAC&ESCC datasets: (**a**) GO BP; (**b**) GO CC enrichment results. Dot size represents gene ratio (number of DEGs associated with a given term), and color intensity reflects adjusted *p*-values (FDR). Only terms with FDR < 0.05 were visualized. EAC-Up: upregulated DEG enrichment results from DEG-EAC dataset; ESCC-Up: upregulated DEG enrichment results from DEG-ESCC dataset; EAC&ESCC-Up: upregulated DEG enrichment results from DEG-EAC&ESCC dataset; EAC-Down: downregulated DEG enrichment results from DEG-EAC dataset; ESCC-Down: downregulated DEG enrichment results from DEG-ESCC dataset; EAC&ESCC-Down: downregulated DEG enrichment results from DEG-EAC&ESCC dataset.

To gain insight into the biological functions and signaling pathways associated with the identified DEGs, we performed Gene Ontology (GO) and Kyoto Encyclopedia of Genes and Genomes (KEGG) enrichment analyses using gene sets from the DEG-EAC, DEG-ESCC, and DEG-EAC&ESCC datasets (Supplementary Tables S1 and S2). Figure 3 and Supplementary Figure S3 illustrate the enrichment patterns of DEGs within each EC subtype, as determined by GO and KEGG pathway analyses. In the upregulated gene sets, the DEG-EAC dataset showed significant enrichment in biological processes (BP) related to extracellular structure and adhesion, including extracellular matrix organization, cell adhesion, and collagen catabolic process, highlighting active remodeling of the tumor microenvironment. Similarly, the DEG-ESCC dataset exhibited enrichment in extracellular matrix organization, cell adhesion, and positive regulation of cell migration, reflecting invasive potential. The DEG-EAC&ESCC dataset, representing genes commonly upregulated in both histological subtypes, was enriched in extracellular matrix organization, collagen fibril organization, and collagen catabolic process. In the downregulated gene sets, the DEG-EAC dataset was significantly enriched in keratinocyte differentiation and keratinization. The DEG-ESCC dataset showed similar patterns, with additional enrichment in fatty acid metabolic processes. Likewise, the DEG-EAC&ESCC dataset demonstrated strong downregulation of keratinocyte differentiation and keratinization. KEGG pathway analysis of upregulated genes revealed that the DEG-EAC dataset was significantly enriched in focal adhesion, PI3K-Akt signaling pathway, and ECM–receptor interaction. The DEG-ESCC dataset was similarly enriched in focal adhesion and ECM–receptor interaction, with additional enrichment in the IL-17 signaling pathway. Notably, the DEG-EAC&ESCC dataset demonstrated strong enrichment in ECM–receptor interaction, focal adhesion, and proteoglycans in cancer. In contrast, downregulated genes yielded fewer significant pathways. The DEG-EAC dataset was enriched in metabolic pathways, while no significant KEGG pathway enrichment was observed in the DEG-ESCC or DEG-EAC&ESCC datasets.

### 2.3. Protein–Protein Interaction Network Construction and Identification of Hub Genes

**Figure 4:**
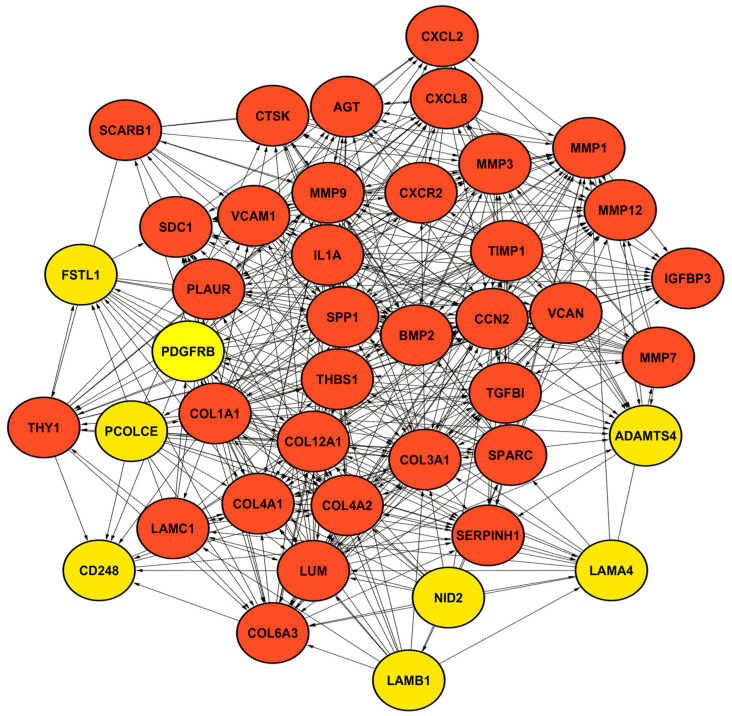
PPI networks of the top-ranked MCODE clusters (Cluster 1) from the DEG-EAC dataset. Red-colored nodes represent hub genes. Yellow-colored nodes represent non-hub genes within the same MCODE cluster.

Protein–protein interaction (PPI) networks were constructed based on the DEG-EAC, DEG-ESCC, and DEG-EAC&ESCC datasets to identify central regulatory genes and functional modules. Candidate hub genes were defined as the top 10% of genes ranked by degree centrality using the CytoHubba plugin in Cytoscape, with degree cut-off values of ≥26 for the DEG-EAC dataset, ≥54 for the DEG-ESCC dataset, and ≥18 for the DEG-EAC&ESCC dataset. In addition, MCODE was employed to conduct clustering analysis on each network. A total of 12, 10, and 3 clusters were identified in the DEG-EAC, DEG-ESCC, and DEG-EAC&ESCC datasets, respectively. These clusters represent the most densely connected modules in each PPI network, and the top MCODE Cluster 1 for each dataset is visualized in Figure 4 and Supplementary Figure S4, where red-colored nodes denote genes ranked in the top 10% by degree centrality. Finally, genes that met both the degree centrality threshold and MCODE score criterion (score > 3) were defined as final hub genes. As a result, 79, 59, and 17 hub genes were identified in the DEG-EAC, DEG-ESCC, and DEG-EAC&ESCC datasets, respectively. The complete lists of identified hub genes are provided in Supplementary Table S4. Furthermore, we identified GO and KEGG terms enriched by the selected hub genes to characterize their biological functions. Detailed information is provided in Supplementary Table S5.

### 2.4. Survival and Expression Analysis of Hub Genes in TCGA Dataset

**Figure 5:**
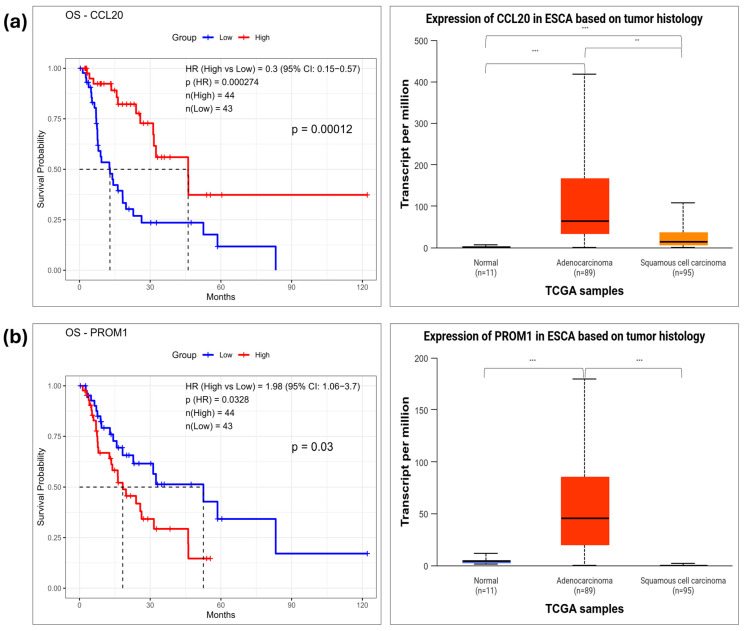
Prognostic significance and expression patterns of survival-associated hub genes in the TCGA-ESCA dataset. Kaplan–Meier survival curves (**left**) and boxplots of mRNA expression (**right**) for seven hub genes from the DEG-EAC dataset. Higher expression of (**a**) CCL20 was associated with improved overall survival, while (**b**) PROM1 expression was linked to poorer prognosis. Statistical significance in the boxplots was annotated as follows: *p*  <  0.01 (**), and *p*  <  0.001 (***).

To evaluate the prognostic relevance of hub genes, OS analysis was conducted using the TCGA-ESCA cohort, which includes 87 EAC and 94 ESCC cases. In the DEG-EAC dataset, seven hub genes were significantly associated with OS. Higher expression of DSP (*p* = 0.0025), DSC3 (*p* = 0.0268), DSC2 (*p* = 0.0484), CCL20 (*p* = 0.00027), MMP12 (*p* = 0.00499), and IL1A (*p* = 0.0094) correlated with improved survival, while high expression of PROM1 (*p* = 0.0328) was linked to lower probability of survival compared to low expression (Figure 5 and Supplementary Figure S5).

**Figure 6:**
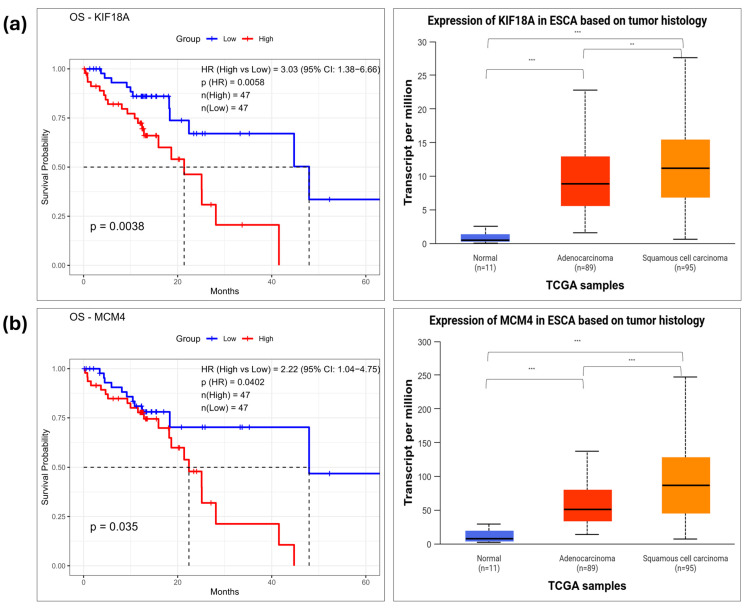
Prognostic significance and expression patterns of survival-associated hub genes in the TCGA-ESCA dataset. Kaplan–Meier survival curves (**left**) and boxplots of mRNA expression (**right**) for seven hub genes from the DEG-ESCC dataset: High expression of (**a**) KIF18A, (**b**) MCM4 was significantly associated with worse overall survival. Statistical significance in the boxplots was annotated as follows: *p*  <  0.01 (**), and *p*  <  0.001 (***).

In the DEG-ESCC dataset, six hub genes were significantly associated with low survival rate. High expression of CHEK1 (*p* = 0.019), KIF18A (*p* = 0.0038), MCM4 (*p* = 0.035), MCM10 (*p* = 0.02), TPX2 (*p* = 0.0084), and TRIP13 (*p* = 0.035) was significantly associated with low survival rate compared to low expression (Figure 6 and Supplementary Figure S6).

In the DEG-EAC&ESCC dataset, no up- and downregulated hub genes were significantly associated with overall survival.

To investigate transcriptional differences in prognostically significant hub genes, mRNA expression levels were examined across normal, EAC, and ESCC tissues using the UALCAN database. In the DEG-EAC dataset, seven hub genes—DSP, DSC3, DSC2, CCL20, MMP12, IL1A, and PROM1—were analyzed. Among them, CCL20, MMP12, IL1A, and PROM1 were significantly overexpressed in EAC tissues compared to normal samples. DSP, DSC3, and DSC2 did not show significant expression differences in EAC. In the DEG-ESCC dataset, six hub genes—CHEK1, TPX2, TRIP13, MCM4, MCM10, and KIF18A—were significantly overexpressed in ESCC tissues compared to normal tissues, supporting their role in ESCC-specific tumorigenesis. These results suggest their potential as shared prognostic biomarkers across histological subtypes of esophageal cancer. Detailed results of survival and expression analysis results for all tested hub genes are provided in Supplementary Table S6 and Supplementary Table S7, respectively.

### 2.5. Computational Screening of Potential Drug Candidates for EC

**Figure 7:**
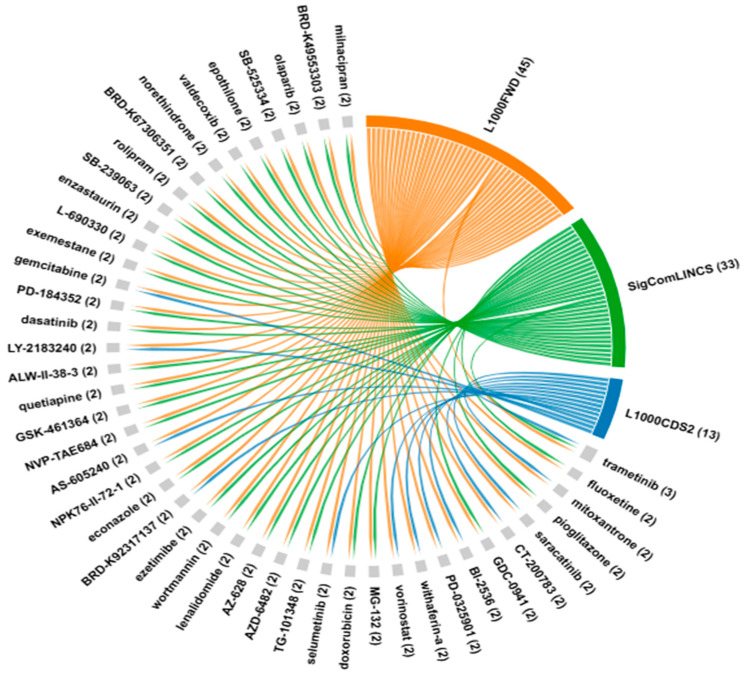
Chord diagrams summarize the potential compounds identified across the three platforms (L1000FWD, L1000CDS2, SigCom LINCS) for the DEG-EAC dataset. Each line connects a platform to its identified compound. Blue lines indicate compounds identified by L1000CDS2, green lines by L1000FWD, and yellow lines by L1000FWD.

To screen the potential drugs to therapeutically target identified DEGs, we conducted screening using all three platforms. Using the L1000FWD platform, 2363 compounds were identified for the DEG-EAC dataset, 2355 for the DEG-ESCC dataset, and 2379 for the DEG-EAC&ESCC dataset. L1000CDS2 analysis identified potential therapeutic compounds, ranked by their overlap scores. Specifically, 42 compounds were identified for the DEG-EAC dataset, 37 for the DES-ESCC dataset, and 42 for the DEG-EAC&ESCC dataset. SigCom LINCS analysis generated two distinct compound lists (100 reversers and 100 mimickers) for each DEG dataset. After removing the duplicate entries, 97, 71, and 96 unique reverser compounds were retained for the DEG-EAC, DEG-ESCC, and DEG-EAC&ESCC datasets, respectively. The common compounds reversing EC-associated gene expression were identified through chord diagram across the three platforms. In the DEG-EAC dataset, the MEK inhibitor “trametinib” was commonly identified across all three platforms (Figure 7). In the DEG-ESCC dataset, six compounds were identified: the MEK inhibitors “PD-0325901”, “selumetinib”, and “trametinib”, the ALK inhibitors “NVP-TAE684”, and PI3K inhibitor “wortmannin”, and the IGF-1 receptor inhibitor “BMS-536924” (Supplementary Figure S7a). In the DEG-EAC&ESCC dataset, the MEK inhibitor “selumetinib” was identified. Trametinib was identified across all three platforms in both the DEG-EAC and DEG-ESCC datasets (Supplementary Figure S7b). Selumetinib was also identified in the DEG-ESCC and DEG-EAC&ESCC datasets. Overlapping compounds identified from pairwise comparisons between the three platforms (L1000FWD, L100CDS2, and SigCom LINCS) for each DEG dataset (DEG-EAC, DEG-ESCC, and DEG-EAC&ESCC) were shown in detail in Supplementary Table S8. No common compounds were identified across the three platforms in all DEG datasets. However, a compound identified in the three-platform overlaps of the DEG-ESCC and DEG-EAC&ESCC datasets was also found in two platforms in the DEG-EAC dataset. Selumetinib and trametinib were identified in the L1000FWD and L1000CDS2 overlap for the DEG-EAC dataset and DEG-EAC&ESCC datasets, respectively.

## 3. Discussion

The current study employed a multilayered, subtype-stratified framework, in recognition of the pronounced biological divergence between EAC and ESCC, that integrated differential gene expression profiling, pathway enrichment analysis, protein–protein interaction-based hub gene prioritization, and survival correlation.

Through the analysis of the DEG-EAC dataset, which boasts a large sample size, numerous hub genes were identified as prognostically informative in EAC. Sixteen of these novel genes had not been previously reported to be relevant to EAC development. Among these genes, CALML4, PKP2, SCARB1, and SERPINH1 showed significant gene expression in one EAC sample compared to a normal sample. In previous studies, highly expressed CALML4 was reported as activating CGMP-PKG signaling pathway and suppressing tumors in gastric cancer [16]. Nevertheless, in EAC samples, upregulated CALML4 was predominantly shown in Rap1, Ras signaling pathway, kaposi sarcoma-associated herpesvirus infection, and phosphatidylinositol signaling system, which was closely related to tumor cell invasion, cancer cell proliferation, and uncontrolled cell metastasis [17,18,19]. In addition, highly expressed PKP2 was also notified in cytoskeleton in muscle cells, cell junction, protein binding, and sodium channel regulator activity in the current study. Previously, PKP2 was revealed to be highly expressed in various cancer types such as lung adenocarcinoma or gastric cancer [20,21], which was consistent with our results. As shown in prior studies, highly expressed SCARB1 was found to be related to developing EAC in the present study [22,23,24]. Furthermore, reported in various cancers except for EAC as relevant to activating PI3K-Akt signaling pathway or promoting tumor through SENP3/SP1/SQLE axis [25,26], SERPINH1 was also identified as a key gene in EAC in the current study. Collectively, the findings from this study—which uniquely identify highly expressed genes in EAC samples compared to normal cells—offer compelling evidence for their potential targets in EAC treatment. In addition, through the present analysis, DSP, DSC3, DSC2, CCL20, MMP12, and IL1A were identified as a central hub gene with prognostic value. Desmocollin 2 and 3 (DSC2 and DSC3) expressions are well-known prognostic indicators in various cancers, related to adhesive strength, cytoskeletal arrangement, and cell adhesion [27,28]. However, specifically concerning tumor promotion, loss of DSC2 expression has only been reported to affect cell proliferation, invasion, tumor differentiation, and even metastasis in ESCC, one of the EC subtypes [29]. Acknowledging the varied expression of DSC2 across diverse cancer types and their respective subtypes, the significant association observed in this study between a low expression of DSC2 in EAC samples (relative to normal tissue) and an improved survival rate, positions DSC2 as a compelling candidate. Still, through analysis of DEG-ESCC dataset, we only found novel genes already reported in prior studies such as CHEK1, TPX2, MCM4, MCM10, TRIP13, and KIF18A in ESCC. Despite these consistent findings of key genes in ESCC, however, the discrepancies in novel genes observed between EAC and ESCC samples might provide evidence of distinct potential as prognostic biomarkers or therapeutic targets, specific to each EC subtype.

In this cross-subtype analysis of esophageal cancer, we identified a set of hub genes that are commonly upregulated in both EAC and ESCC. These shared hub genes—which include multiple collagens (e.g., COL1A1, COL1A2, COL3A1), extracellular matrix (ECM) glycoproteins (SPARC, THBS1, TGFBI), proteoglycans (BGN, LUM, VCAN), and matrix remodelers (MMP1, MMP9, TIMP1, SPP1, POSTN)—underscore a convergent biology centered on tumor–stroma interactions in both histological subtypes. The ubiquitous dysregulation of these genes in both EAC and ESCC highlights their fundamental role in esophageal tumor biology. Functional enrichment analysis of the shared dysregulated genes strongly supports their involvement in ECM organization and cell adhesion processes, which suggests to drive tumor progression in both EAC and ESCC [30,31]. COL isoforms including COL1A1, COL1A2, and COL3A1 were previously reported as prominent hub genes related to collagen deposition and reorganizing. A single-center study reported COL isoforms were accumulated in ECM in ESCC patient samples since collagen is a crucial protein in ECM [32,33]. Furthermore, type I collagen secreted by ESCC was closely related to tumor progression, invasion, and potentially poor prognosis, which often correlates with treatment resistance [34]. Yang et al. demonstrated that fibroblast-derived collagen I promotes radioresistance in ESCC by enhancing DNA damage repair in tumor cells and inducing a CXCL1–CXCR2 paracrine loop that further activates cancer-associated fibroblasts [35]. Even so, while few studies have reported COL isoforms in EAC, or identified them as shared hub genes in both EAC and ESCC, the current analysis offers crucial insights into potential targets for both EC subtypes. In addition, we also found upregulation of MMP family members (MMP1, MMP9) and the inhibitor TIMP1 among the shared hubs, indicating active matrix turnover. MMP1 is an enzyme that degrades ECM components, including collagen, playing a vital role in promoting cancer cell invasion, metastasis, and angiogenesis within the tumor microenvironment [36]. Particularly in EC, and more specifically in ESCC, increased expression of MMP1 is frequently observed. This elevation is closely associated with tumor growth, invasion, lymph node metastasis, and poor patient prognosis [37]. MMP1 degrades the ECM, removing physical barriers that impede cancer cell migration into surrounding tissues and infiltration into blood and lymphatic vessels, ultimately accelerating cancer metastasis [38]. Furthermore, the MMP-1/PAR-1 (Protease-Activated Receptor-1) signaling axis plays a crucial role in tumorigenesis in ESCC, suggesting its potential as a novel therapeutic target. MMP9 is also a potent type IV collagenase that facilitates invasion. Tsukamoto et al. reported that MMP9 is induced at the invasive front of ESCC tumors through direct contact with infiltrating M2 macrophages, and that high cancer-cell MMP9 expression correlates with significantly worse patient survival (*p* = 0.03) [39]. Thus, even though MMP9 was not prognostic in our dataset, its known role in promoting invasion and metastasis in ESCC underscores the biological importance of matrix-degrading enzymes in both subtypes. Furthermore, several shared hub genes encode matricellular proteins—secreted factors that modulate cell–ECM interactions and signaling. These include SPP1 (osteopontin), SPARC, and POSTN (periostin), all of which have been implicated in esophageal cancer progression. SPP1 is a pro-metastatic factor and was identified as a hub gene in an integrative analysis of esophageal carcinoma. Cai et al. consistently found that SPP1 is markedly overexpressed in esophageal tumors (both EAC and ESCC) and associated with advanced stage and lymph node metastasis; functionally, SPP1 drives tumor cell migration and invasion by activating the focal adhesion kinase (FAK)–ERK signaling pathway [40]. Targeting this pathway (for instance, using a FAK inhibitor) was shown to suppress esophageal cancer cell invasiveness, highlighting SPP1’s role in mediating aggressive phenotypes. Another shared key gene is SPARC (secreted protein acidic and rich in cysteine), a matricellular protein that influences ECM assembly and growth factor signaling. SPARC was a common upregulated hub gene in our analysis and appears to originate predominantly from the tumor stroma. In EAC, high stromal SPARC levels have been linked to tumor progression: Ma et al. reported that SPARC expression in the tumor microenvironment correlates with worse disease-specific survival in EAC patients [41]. SPARC could cooperate with TGF-β signaling to induce partial epithelial-to-mesenchymal transition (EMT) in EAC cells, thereby enhancing their invasiveness [41]. This finding aligns with the idea that cancer-associated fibroblasts and other stromal cells (which secrete SPARC) can actively promote a more mesenchymal, motile phenotype in esophageal adenocarcinoma epithelium. POSTN (Periostin), an ECM glycoprotein induced by TGF-β, emerged as one of the top hub genes and exemplifies the tumor–stroma crosstalk common to EAC and ESCC. Periostin is not normally produced by esophageal epithelial cells, but rather by activated fibroblasts in the tumor stroma (i.e., cancer-associated fibroblasts, CAFs). Elevated POSTN in the tumor microenvironment has been documented in both subtypes. In ESCC, Miyako et al. demonstrated that CAF-secreted periostin promotes cancer progression by enhancing the migratory and survival capacity of tumor cells and by recruiting stromal cells [42]. High periostin expression in ESCC patient tumors was strongly associated with deeper invasion, lymphovascular spread, and a higher pathologic stage, as well as greater influx of CAFs and M2 macrophages. Clinically, ESCC patients with periostin-rich stroma had significantly poorer postoperative outcomes. These data firmly establish periostin as a pro-tumor effector in ESCC [42]. Intriguingly, a similar paradigm is observed in EAC: CAF-derived periostin can bind to integrin receptors on EAC cells and activate PI3K–Akt signaling to drive invasion [43,44]. Indeed, targeting the periostin–integrin axis has been suggested as a strategy to impede tumor–stromal cooperation in esophageal cancer. The prominence of POSTN, SPARC, and TGFBI among the shared hubs also underscores the role of TGF-β pathway activity and a myofibroblastic stroma in both tumor types.

Based on the prognostic biomarkers found through the analysis of each dataset, DEG-EAC, DEG-ESCC, and DEG-EAC&ESCC, in the current study, we identified matched potential compounds. The effectiveness of these platforms has been demonstrated in various therapeutic areas. Three platforms to identify potential compounds targeted to the prognostic biomarkers have been applied across diverse areas, including cancer research (e.g., lung adenocarcinoma [45], breast cancer [46], and acute lymphoblastic leukemia [47]), neurodegenerative and chronic diseases (e.g., Alzheimer’s disease [48] and steroid-induced osteonecrosis [49]), and acute conditions (e.g., brain injury [50], acute liver failure [51], and COVID-19 [52]). The present drug repurposing analysis suggested selumetinib and trametinib as potential therapeutic candidates for each and both EC subtypes. As a second-generation, selective, potent, and non-ATP competitive allosteric MEK (mitogen-activated protein kinase kinase) 1/2 inhibitor, the current study also suggested potential target disease, EC, to use selumetinib and trametinib. MEK is a critical component of the MAPK (mitogen-activated protein kinase) signaling pathway. It becomes activated upon phosphorylation by upstream Raf, which in turn phosphorylates and activates its downstream target, ERK (extracellular signal-regulated kinase). Activated ERK translocates to the nucleus, where it modulates the activity of various transcription factors, including AP-1 (activator protein-1), known to bind to the promoter region of the MMP family, such as the MMP1 and MMP9 genes, shown as shared genes of both EC subtypes in the current study [53]. Consistently, prior studies suggested MEK/ERK signaling pathway plays a critical role in promoting the proliferation, invasion, and migration of EC cells interacting with MMP1 and MMP9 expression [54,55]. Although selumetinib was approved for neurofibromas in many countries including FDA, one of potential MEK inhibitors and treatment options, selumetinib still has been investigated various solid tumors such as colorectal cancer, lung cancer, and gastric cancers including EC. Currently, phase 1 trial showed 30% target size reduction of EC after using combination between selumetinib and pembrolizumab, which was not, nevertheless, considered as partial response [56]. Since a prior phase II study demonstrated the efficacy and safety of selumetinib plus docetaxel as second-line therapy in advanced gastric cancer patients showing RR was noted as 28.0% (95 CI 0.12–0.49) without grade 3 or 4 AE, considering our analysis result, expanded clinical studies are needed, including more EC patients [57]. In addition, as a type III allosteric, non-ATP competitive, and highly selective inhibitor of MEK1/2, trametinib has been approved by the FDA for the treatment of several BRAF V600E-mutated cancers, including metastatic non-small cell lung cancer, metastatic melanoma (in combination with dabrafenib), and locally advanced or metastatic anaplastic thyroid cancer [58]. In a previous study investigating the chemoresistance mechanism in EAC, MEK inhibitors including selumetinib and trametinib were shown to enhance cisplatin-induced cytotoxicity when combined with IGFBP2 knockdown, which reflects that MEK signaling could be a promising strategy to overcome this resistance depending on the tumor microenvironment [59]. In vivo study also demonstrated trametinib effectively suppressed tumor growth and prolonged median survival in ESCC. In clinical studies, the efficacy of trametinib could not be demonstrated with any significant improvement of treatment response in EC patients [60,61]. However, it is necessary to consider that pathological conditions may give rise to new undesirable side effects due to the action on other proteins not expressed in control conditions or in the pathologies of previous clinical trials with MEK inhibitors such as selumetinib and trametinib. Trametinib has been reported to induce cardiotoxicity by disrupting the balance of signaling pathways like IL-6/PI3K/AKT in diseased heart tissue—pathways that may not be active in healthy controls. Gilbert et al. reported that approximately 11% of patients treated with trametinib experienced a reduction in ejection fraction of >10%, as determined by echocardiography, which was consistently supported by a current report recommending monitoring cardiotoxicity of patients treated with MEK inhibitors in the first year [62,63]. Similarly, selumetinib has been associated with adverse responses in inflammatory settings, where it can affect additional molecular processes not previously observed [64]. These side effects are particularly concerning because they may not be predicted from trials conducted under controlled or limited pathological contexts. Therefore, since selumetinib and trametinib were only tested in early-phase clinical trials with a limited number of EC patients, further studies are needed to fully evaluate the efficacy and safety of MEK inhibitors as potential drugs in various settings, particularly considering the hub genes and matched compounds identified in the current research.

This study has several limitations. Analyses were based on bulk transcriptomic datasets and in silico drug prediction platforms, which cannot account for intratumoral heterogeneity or drug bioavailability. Among these, the L1000CDS2 platform provides only the top 50 drug candidates ranked by overlap score, which limits the scope of compound identification. To compensate for this, we combined results from three complementary screening platforms to enhance the reliability of drug repurposing. In addition, the current analysis did not include betweenness and closeness centrality for identifying candidate hub genes. To avoid bias, employing these centrality methodologies is valuable. Although betweenness and closeness centrality are important from a network topology perspective, they primarily reflect information flow or shortest paths between nodes—concepts rooted in graph theory rather than biological function. In our preliminary analysis, we also observed that integrating multiple centrality measures often yielded conflicting results and excluded several biologically meaningful and prognostically relevant genes. Thus, the current study adopted a strategy that first identifies highly interactive nodes through degree centrality—reflecting the extent of direct molecular interactions—and then applies MCODE clustering to detect functionally coherent gene modules within the PPI network [65,66,67,68]. Future work should include single-cell analyses, proteomic integration, and functional validation in patient-derived models to refine subtype-specific precision therapies. In addition, the current study did not include further validation methods to identify compounds or hub genes such as molecular docking and dynamic simulations. Although molecular docking and dynamics simulations are valuable for validating compound–target interactions, the scope of this study was focused on transcriptomic signature reversal using large-scale expression datasets. Molecular docking and dynamic simulations could not be included in our workflow because several hub genes (e.g., DSC3, PROM1) identified in our analysis do not have well-characterized 3D structures in the Protein Data Bank (https://www.rcsb.org/, accessed on 3 August 2025), which was similarly noted as limitations in prior studies [69,70,71]. To be consistent, approaches that use other types of molecular data (e.g., proteomics, which reflects the actual expression of proteins encoded by the proposed genes) or molecular simulations should also be integrated to validate the efficacy of the proposed drugs for new therapeutic targets, or to assess whether the identified genes may undergo mutations in the pathological conditions of interest, potentially altering the effectiveness of protein–drug interactions. In particular, validation through clinical trials will ultimately be required. Furthermore, we could not evaluate prognostic biomarkers alongside other clinical characteristics like ethnicity, age, or sex due to limited access to open-source clinical datasets with diverse demographic information. Future studies will explore these factors once we gain approval to access relevant national databases.

In summary, our comprehensive and subtype-stratified approach identifies hub genes with prognostic and therapeutic relevance in EC subtypes, and proposes MEK inhibitors as promising candidates for drug repurposing. This work lays a robust foundation for future research in precision oncology for esophageal cancer. Nevertheless, alongside undesirable side effects, additional validation incorporating proteomic, mutational, and pharmacodynamic data, as well as preclinical and clinical studies, is necessary to confirm the translational relevance of the proposed targets and compounds.

## 4. Materials and Methods

### 4.1. Data Acquisition

To identify DEGs, publicly available gene expression datasets were retrieved from the GEO database (https://www.ncbi.nlm.nih.gov/geo/, accessed on 16 July 2025). For EAC, the GSE13898 [72] and GSE92396 datasets were used, while GSE38129 [73], GSE44021 [74], and GSE161533 were utilized for ESCC. Additionally, the GSE26886 dataset [75], which includes gene expression data from both EAC and ESCC samples, was incorporated to explore shared molecular features between the two subtypes, EAC and ESCC. In addition, clinical and RNA sequencing data for esophageal cancer patients were obtained from the TCGA-ESCA cohort via the cBioPortal database (https://www.cbioportal.org/, accessed on 16 July 2025) [76].

### 4.2. Identification of Common DEGs Based on Esophageal Cancer Subtypes

DEGs were identified using GEO2R (https://www.ncbi.nlm.nih.gov/geo/geo2r/, accessed on 16 July 2025), an online analysis tool provided by GEO [77]. Each dataset was independently analyzed by comparing tumor and matched normal samples. The *p*-values were adjusted using the Benjamini–Hochberg false discovery rate (FDR) method, and data were log-transformed using the auto-detect option. Genes with an adjusted *p*-value < 0.05 and |log2 fold change| > 1 were considered significant DEGs. To identify subtype-specific and commonly found DEGs in EAC and/or ESCC datasets, three distinct DEG datasets were derived through comparison with normal tissues: the DEG-EAC dataset (identifying DEGs within EAC samples), the DEG-ESCC dataset (identifying DEGs within ESCC samples), and the DEG-EAC&ESCC dataset (comprising DEGs commonly found in both EAC and ESCC samples). These DEG datasets were used in downstream analyses including functional enrichment, network construction, and survival analysis. To extract robust and consistently dysregulated genes for each cancer subtype, Venn diagram analyses were performed to identify genes commonly upregulated or downregulated across the datasets. Gene overlaps were visualized using the EVenn tool (https://www.bic.ac.cn/EVenn/#/, accessed on 16 July 2025) [78].

### 4.3. Functional and Pathway Enrichment Analysis

GO and Kyoto Encyclopedia of Genes and Genomes (KEGG) pathway enrichment analyses were performed using DAVID (https://david.ncifcrf.gov/, accessed on 16 July 2025) [79]. GO analysis encompassed BP, molecular functions (MF), and cellular components (CC). KEGG pathway analysis was used to investigate molecular interaction networks. We also evaluated enrichment patterns based on EC subtypes using GO and KEGG pathway analysis, alongside individual gene analysis. Enrichment results with *p*-value < 0.05 were considered statistically significant. To visualize the distribution of enriched gene counts and the statistical significance (adjusted *p*-values) of GO terms and KEGG pathways, boxplots were generated using the ggplot2 package in R (version 4.4.1).

### 4.4. Protein–Protein Interaction Network Analysis

PPI networks were constructed using STRING (https://string-db.org, accessed on 16 July 2025) [80] with a minimum required interaction score of 0.4. The CytoHubba plugin (version 0.1) [81] in Cytoscape (version 3.10.3) [82] was used to calculate the degree score of each gene in the network. Genes ranking in the top 10% by degree centrality were considered as candidate hub genes. MCODE analysis was conducted using the MCODE plugin (version 2.0.3) [83] in Cytoscape with the following parameters: MCODE score > 3, degree cut-off = 2, node score cut-off = 0.2, maximum depth = 100, and k-score = 2. Genes that were ranked in the top 10% by degree centrality and also located within MCODE clusters with a score greater than 3 were defined as hub genes for further analysis.

### 4.5. Survival and Expression Analysis of Hub Genes

Clinical and RNA sequencing data from the TCGA-ESCA cohort were used for the survival analysis after extracting and splitting datasets based on the types of cancer cells such as EAC and/or ESCC. Survival analyses were conducted using the survival and survminer packages in R (version 4.4.1). Hazard ratios (HRs) and 95% confidence intervals (CIs) were calculated, and *p*-values < 0.05 were considered statistically significant. In addition, the UALCAN database (https://ualcan.path.uab.edu/, accessed on 16 July 2025) was used to evaluate the expression levels of hub genes from the EAC, ESCC, and EAC&ESCC datasets across histological subtypes [84].

### 4.6. Drug Repurposing Analysis Based on the Esophageal Cancer Subtypes

Through analyzing three different DEG datasets (DEG-EAC, DEG-ESCC, and DEG-EAC&ESCC), we identified L1000 small molecule signatures that match user input signatures (i.e., upregulated and downregulated genes) with three online platforms such as L1000FWD, L1000CDS2, and SigCom LINCS. These platforms are integral components of the NIH library of integrated network-based cellular signatures program, and each unique data and analytical capabilities to support drug discovery [85]. L1000FWD is a LINCS-based platform that provides transcriptomic profiles of human cell lines in response to over 20,000 small molecule compounds, enabling similarity-based comparison of drug-induced gene expression signatures [86]. L1000FWD calculates a similarity score ranging from −1 to +1, where positive scores indicate similar gene expression patterns and negative scores indicate opposite (reversal) patterns between input signatures and drug-induced signatures. L1000CDS2 processes over 1 million gene expression profiles using the characteristic direction method, which has been shown to outperform other methods in terms of signal-to-noise ratio, allowing the identification of compounds that can reverse disease-associated gene signatures [87]. In L1000CDS2’s reverse mode, compounds are ranked based on their predicted ability to reverse input gene signatures, with only the top 50 drug signatures returned by the platform. SigCom LINCS complements existing drug perturbation platforms by integrating approximately 1.5 million transcriptional signatures from LINCS, GTEx, and GEO, enabling cross-validation through multi-omics data integration and enhancing candidate reliability without reliance on specific cell lines [88]. SigCom LINCS performs the Mann–Whitney U test separately for up and down gene sets to obtain z-scores, determining whether input genes are positioned toward the top or bottom ranks of a signature. Signatures with negative z-sum values are classified as reversers. Compounds with similarity scores < 0 from L1000FWD, top 50 reversers from L1000CDS2, and signatures with negative z-sum from SigCom LINCS were selected as potential therapeutic compounds. These platforms were used to identify two categories of compounds: reversers (compounds that normalize ESCA gene expression patterns toward non-ESCA levels) and mimickers (compounds that further amplify ESCA-associated expression changes). We identified small molecule drugs that displayed an opposite correlation with theses DEGs, thereby identifying reverser compounds. The potential compounds were visualized using a chord diagram developed by RAWGraphs 2.0 (https://www.rawgraphs.io/, accessed on 3 July 2025), illustrating compounds identified in at least two of the three platforms.

## 5. Conclusions

This study applied a comprehensive transcriptome-based approach to identify subtype-specific hub genes in EAC and ESCC, revealing both distinct molecular features and shared tumor–stroma interactions. The integrative analysis suggested potential therapeutic relevance of MEK inhibitors, particularly through their association with ECM remodeling pathways. However, as our findings are based on gene expression data, further investigation incorporating proteomic validation, functional studies, and clinical evaluation is warranted. These results serve as a preliminary step toward precision oncology in esophageal cancer rather than definitive therapeutic guidance.

## Data Availability

The datasets (GSE13898, GSE93296, GSE38129, GSE44021, GSE161533, and GSE26886) analyzed in this current study are available from the Gene Expression Omnibus database repository (https://www.ncbi.nlm.nih.gov/geo/, accessed on 16 July 2025). Other relevant raw data can be provided upon reasonable request.

## Supplementary Materials

The following supporting information can be downloaded at: https://www.mdpi.com/article/10.3390/ph18081181/s1, Figure S1: Volcano plots for comparing ESCC vs. normal samples; Figure S2: Venn diagrams showing overlapping upregulated and downregulated DEGs within the ESCC and DEG-EAC&ESCC datasets; Figure S3: Dot plots showing enriched GO molecular functions and KEGG pathways for each DEG dataset; Figure S4: PPI networks of the top-ranked MCODE clusters (Cluster 1) from the DEG-ESCC and DEG-EAC&ESCC datasets; Figure S5: Kaplan–Meier curves and expression boxplots for survival-associated hub genes in the DEG-EAC dataset; Figure S6: Kaplan–Meier curves and expression boxplots for survival-associated hub genes in the DEG-ESCC dataset; Figure S7: Chord diagrams showing compounds identified across three platforms (L1000FWD, L1000CD2, SigCom LINCS) for DEG-ESCC and DEG-EAC&ESCC datasets, Table S1: Full GO and KEGG enrichment analysis results for upregulated DEGs identified in the DEG-EAC, DEG-ESCC, and DEG-EAC&ESCC datasets; Table S2: Full GO and KEGG enrichment analysis results for downregulated DEGs across DEG-EAC, DEG-ESCC, and DEG-EAC&ESCC datasets; Table S3: MCODE clustering results of PPI networks constructed from DEG-EAC, DEG-ESCC, and DEG-EAC&ESCC datasets, including cluster scores and the list of genes within each cluster; Table S4: Hub genes identified from each dataset (DEG-EAC, DEG-ESCC, and DEG-EAC&ESCC), defined by top 10% degree centrality and inclusion in MCODE modules with scores > 3; Table S5: Functional enrichment results (GO and KEGG) for selected hub genes, highlighting biological processes and signaling pathways significantly associated with each subtype; Table S6: Kaplan–Meier survival analysis results for all hub genes across DEG-EAC, DEG-ESCC, and DEG-EAC&ESCC datasets using the TCGA-ESCA cohort. Includes hazard ratios (HR), confidence intervals, and *p*-values for overall survival (OS) associations; Table S7: Expression analysis of hub genes based on TCGA-ESCA data from the UALCAN portal, comparing tumor and normal tissues across EAC and ESCC subtypes; Table S8: Overlapping small-molecule compounds identified as potential reversers of EC-related gene signatures by at least two of the three drug repurposing platforms—L1000FWD, L1000CDS2, and SigCom LINCS.

## Abbreviations

The following abbreviations are used in this manuscript:

BP	Biological Process
CAF	Cancer-Associated Fibroblast
CC	Cellular Component
CI	Confidence Interval
DEG	Differentially Expressed Gene
EAC	Esophageal Adenocarcinoma
EC	Esophageal Cancer
ECM	Extracellular Matrix
EMT	Epithelial–Mesenchymal Transition
ERK	Extracellular Signal-Regulated Kinase
ESCC	Esophageal Squamous Cell Carcinoma
FAK	Focal Adhesion Kinase
FDA	U.S. Food and Drug Administration
FDR	False Discovery Rate
GEO	Gene Expression Omnibus
GO	Gene Ontology
HR	Hazard Ratio
KEGG	Kyoto Encyclopedia of Genes and Genomes
L1000CDS2	L1000 Characteristic Direction Signature Search Engine
L1000FWD	L1000 Fireworks Display
LINCS	Library of Integrated Network-based Cellular Signatures
MCODE	Molecular Complex Detection
MEK	Mitogen-Activated Protein Kinase Kinase
MF	Molecular Function
OS	Overall Survival
PAR-1	Protease-Activated Receptor-1
PPI	Protein–Protein Interaction
STRING	Search Tool for the Retrieval of Interacting Genes/Proteins
TCGA	The Cancer Genome Atlas
UALCAN	University of Alabama at Birmingham Cancer data analysis portal
