# Supplementary material for: Evaluating Potential Therapeutic Targets and Drug Repurposing Based on the Esophageal Cancer Subtypes
Source: Pharmaceuticals (Basel). 2025 Aug 11;18(8):1181. doi: 10.3390/ph18081181 (PMC12389467; doi:10.3390/ph18081181)
Supplement: Supplementary file 1 [file pharmaceuticals-18-01181-s001.zip › Supplementary Figures S1-S7.pdf]

Supplementary Figures

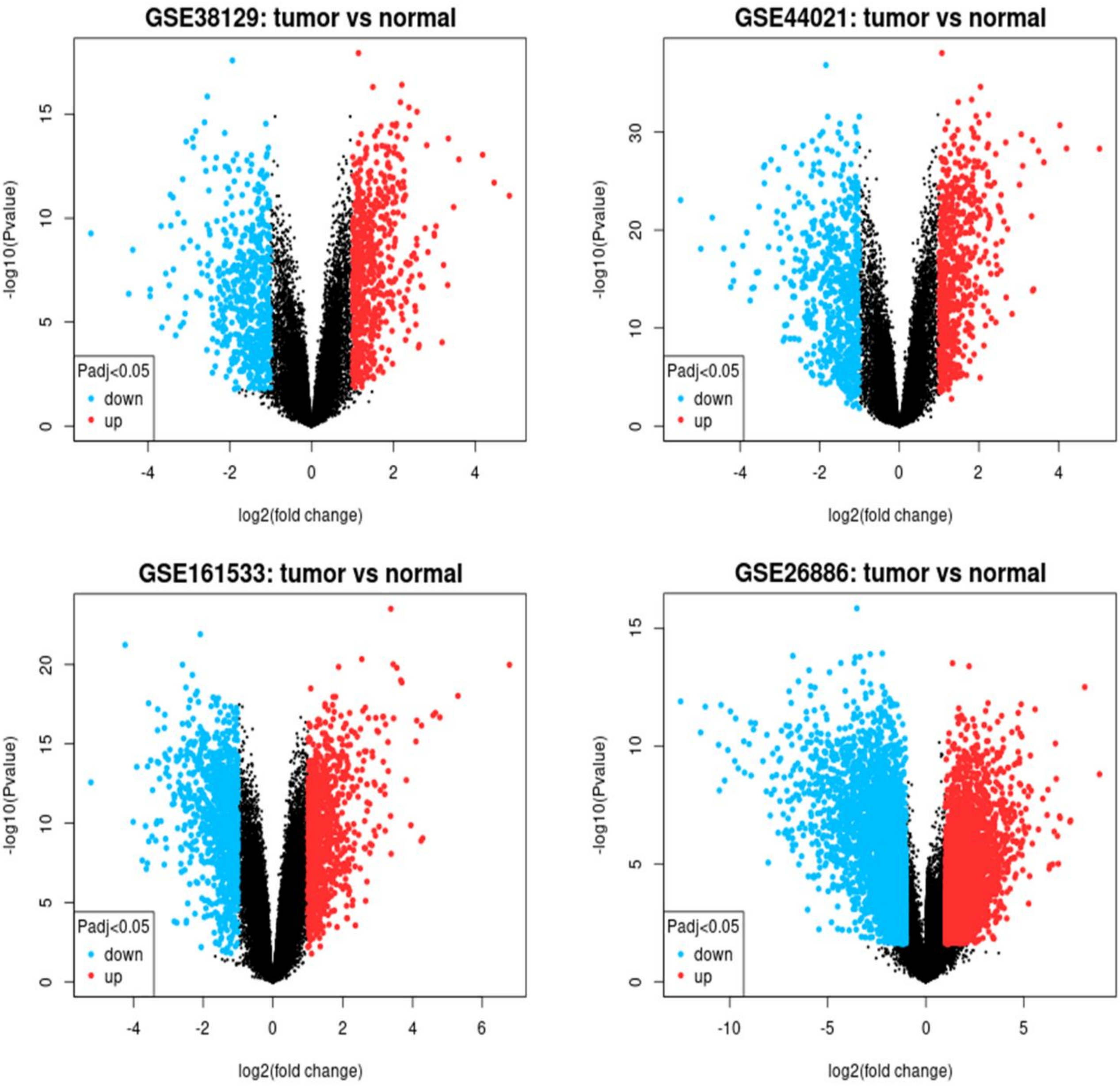

Supplementary Figure S1. Volcano plots for comparing ESCC vs normal samples

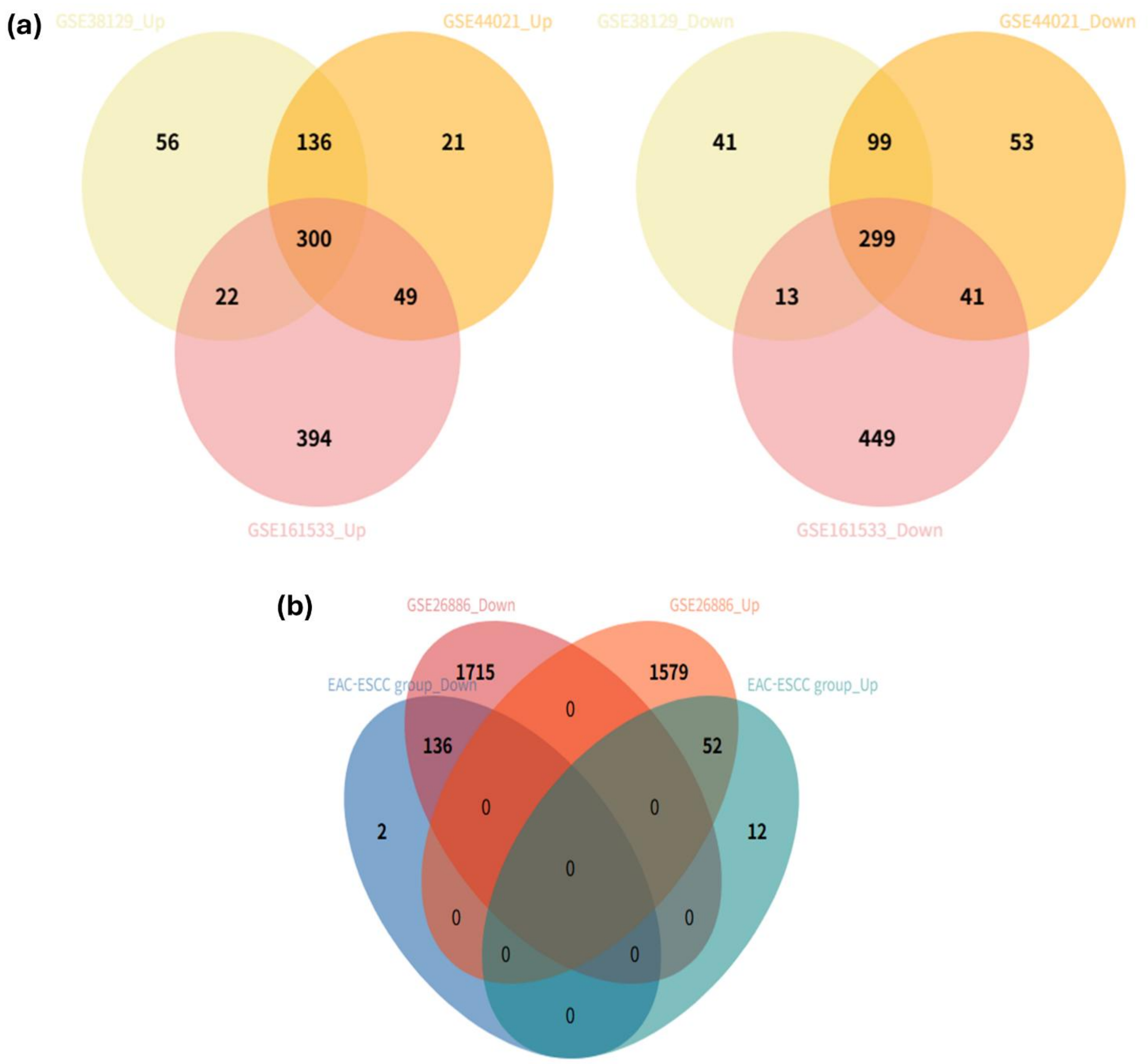

Supplementary Figure S2. Identification of distinct and shared DEGs: (a) Venn diagrams showing overlapping upregulated and downregulated DEGs within the ESCC datasets; (b) Common DEGs shared between EAC and ESCC from GSE26886 and their intersection with other datasets to define the DEG-EAC&ESCC dataset.

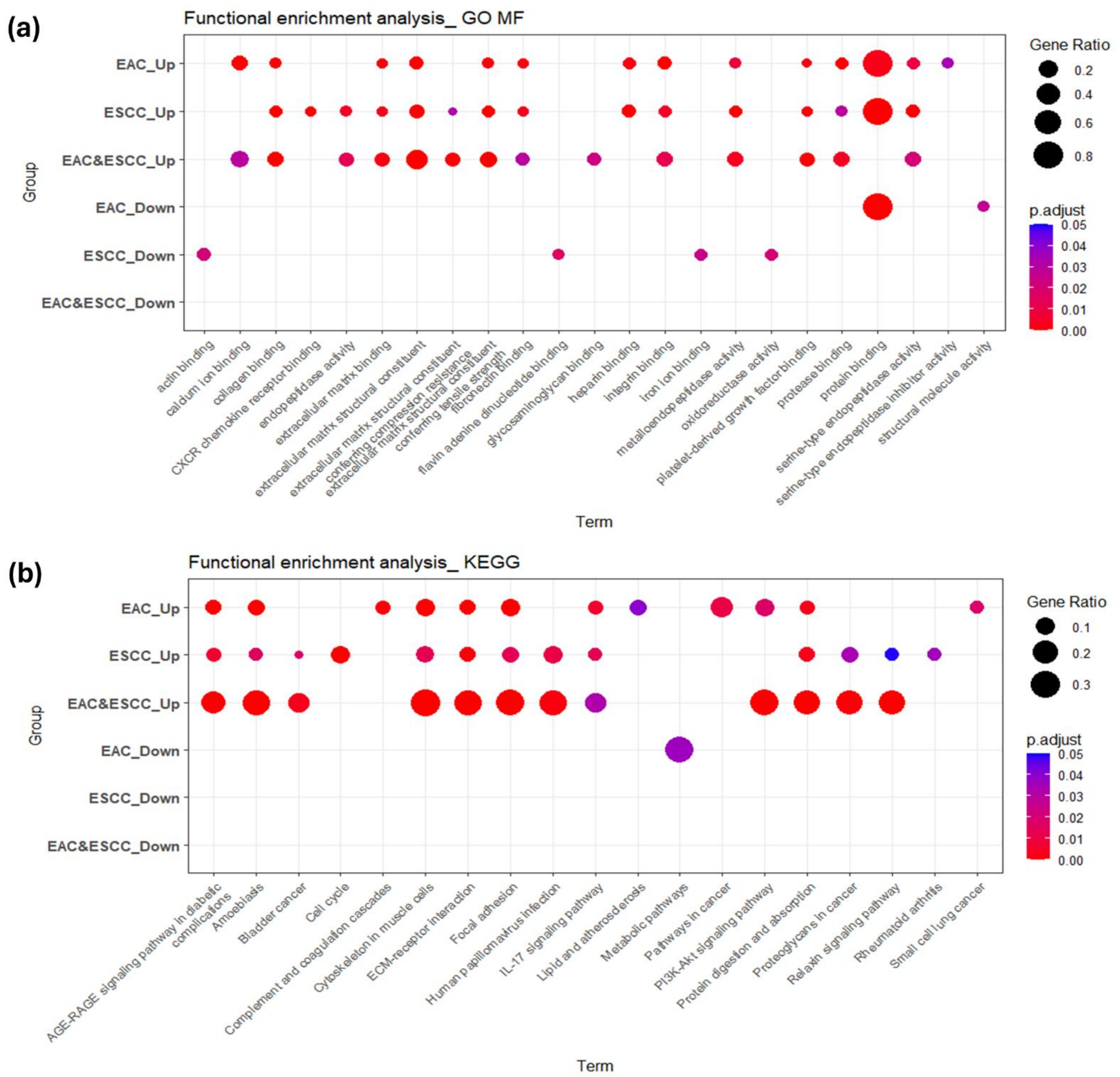

Supplementary Figure S3. Enrichment patterns of DEGs after functional enrichment analysis based on EAC, ESCC, and EAC&ESCC datasets. (a) GO MF; and (b) KEGG pathway enrichment results. Dot size represents gene ratio (number of DEGs associated with a given term), and color intensity reflects adjusted p-values (FDR). Only terms with FDR < 0.05 were visualized. EAC-Up: upregulated DEG enrichment results from DEG-EAC dataset; ESCC-Up: upregulated DEG enrichment results from DEG-ESCC dataset; EAC&ESCC-Up: upregulated DEG enrichment results from DEG-EAC&ESCC dataset; EAC-Down: downregulated DEG enrichment results from DEG-EAC dataset; ESCC-Down: downregulated DEG enrichment results from DEG-ESCC dataset; EAC&ESCC-Down: downregulated DEG enrichment results from DEG-EAC&ESCC dataset

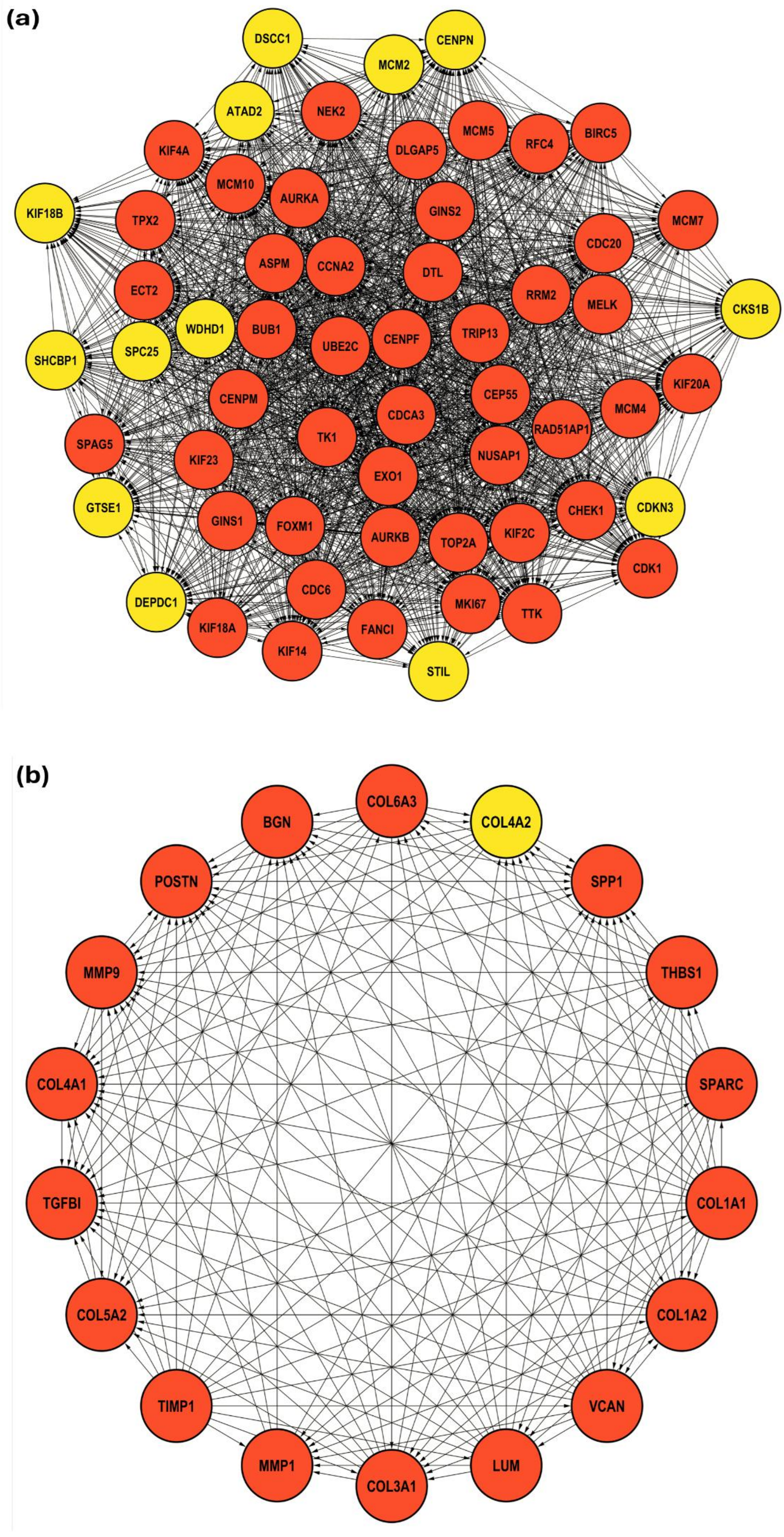

Supplementary Figure S4. PPI networks of the top-ranked MCODE clusters (Cluster 1) from each DEG dataset: (a) DEG-ESCC dataset; (b) DEG-EAC&ESCC dataset. Red-colored nodes represent hub genes. Yellow-colored nodes represent non-hub genes within the same MCODE cluster.

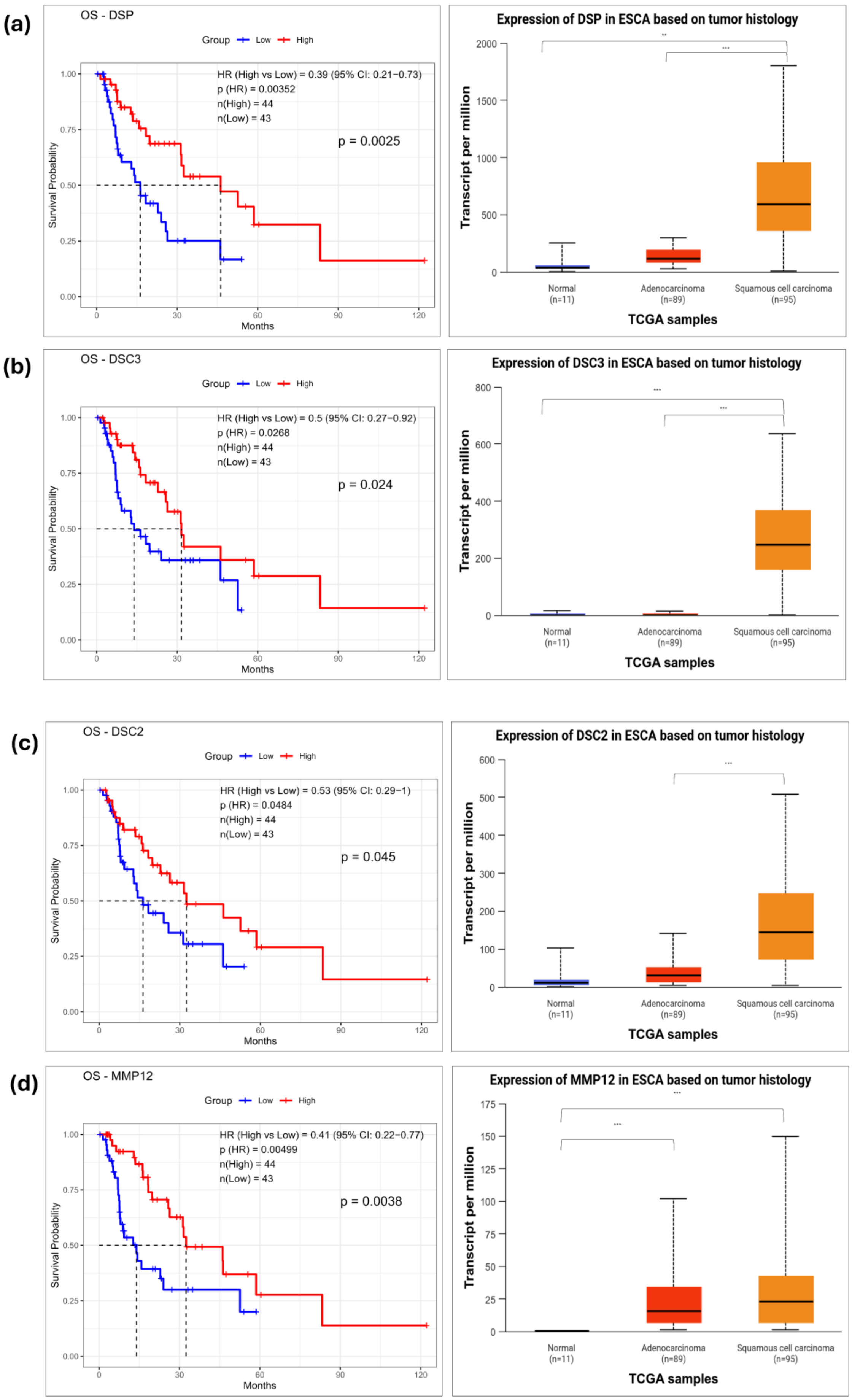

(a)
OS - DSP
Group Low High
HR (High vs Low) = 0.39 (95% CI: 0.21−0.73)
p (HR) = 0.00352
n(High) = 44
n(Low) = 43
p = 0.0025
Survival Probability
Months
Expression of DSP in ESCA based on tumor histology
Transcript per million
Normal (n=11)
Adenocarcinoma (n=89)
Squamous cell carcinoma (n=95)
TCGA samples
(b)
OS - DSC3
Group Low High
HR (High vs Low) = 0.5 (95% CI: 0.27−0.92)
p (HR) = 0.0268
n(High) = 44
n(Low) = 43
p = 0.024
Survival Probability
Months
Expression of DSC3 in ESCA based on tumor histology
Transcript per million
Normal (n=11)
Adenocarcinoma (n=89)
Squamous cell carcinoma (n=95)
TCGA samples
(c)
OS - DSC2
Group Low High
HR (High vs Low) = 0.53 (95% CI: 0.29−1)
p (HR) = 0.0484
n(High) = 44
n(Low) = 43
p = 0.045
Survival Probability
Months
Expression of DSC2 in ESCA based on tumor histology
Transcript per million
Normal (n=11)
Adenocarcinoma (n=89)
Squamous cell carcinoma (n=95)
TCGA samples
(d)
OS - MMP12
Group Low High
HR (High vs Low) = 0.41 (95% CI: 0.22−0.77)
p (HR) = 0.00499
n(High) = 44
n(Low) = 43
p = 0.0038
Survival Probability
Months
Expression of MMP12 in ESCA based on tumor histology
Transcript per million
Normal (n=11)
Adenocarcinoma (n=89)
Squamous cell carcinoma (n=95)
TCGA samples

(e)

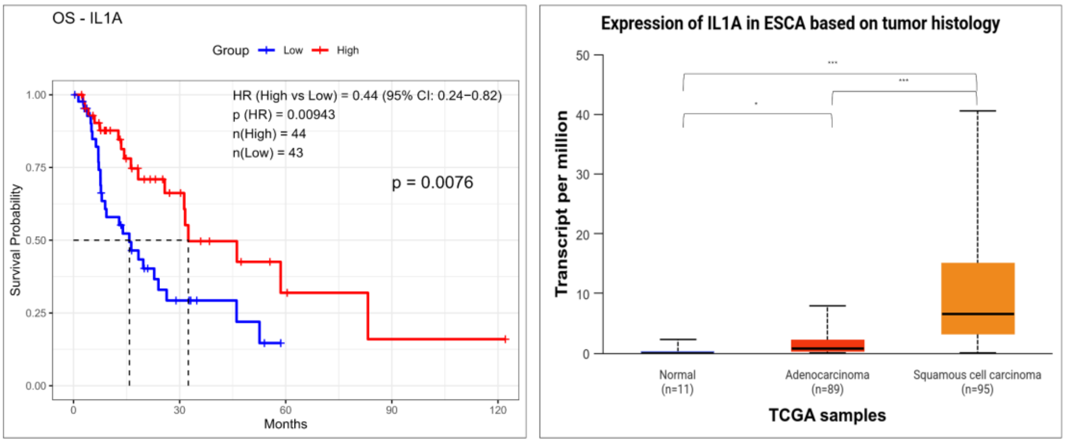

Supplementary Figure S5. Prognostic significance and expression patterns of survival-associated hub genes in the TCGA-ESCA dataset. Kaplan–Meier survival curves (left) and boxplots of mRNA expression (right) for seven hub genes from the DEG-EAC dataset. Higher expression of (a) DSP, (b) DSC3, (c) DSC2, (d) MMP12, and (e) IL1A was associated with improved overall survival. Statistical significance in the box-plots was annotated as follows: $p < 0.05$ (*), $p < 0.01$ (**), and $p < 0.001$ (***).

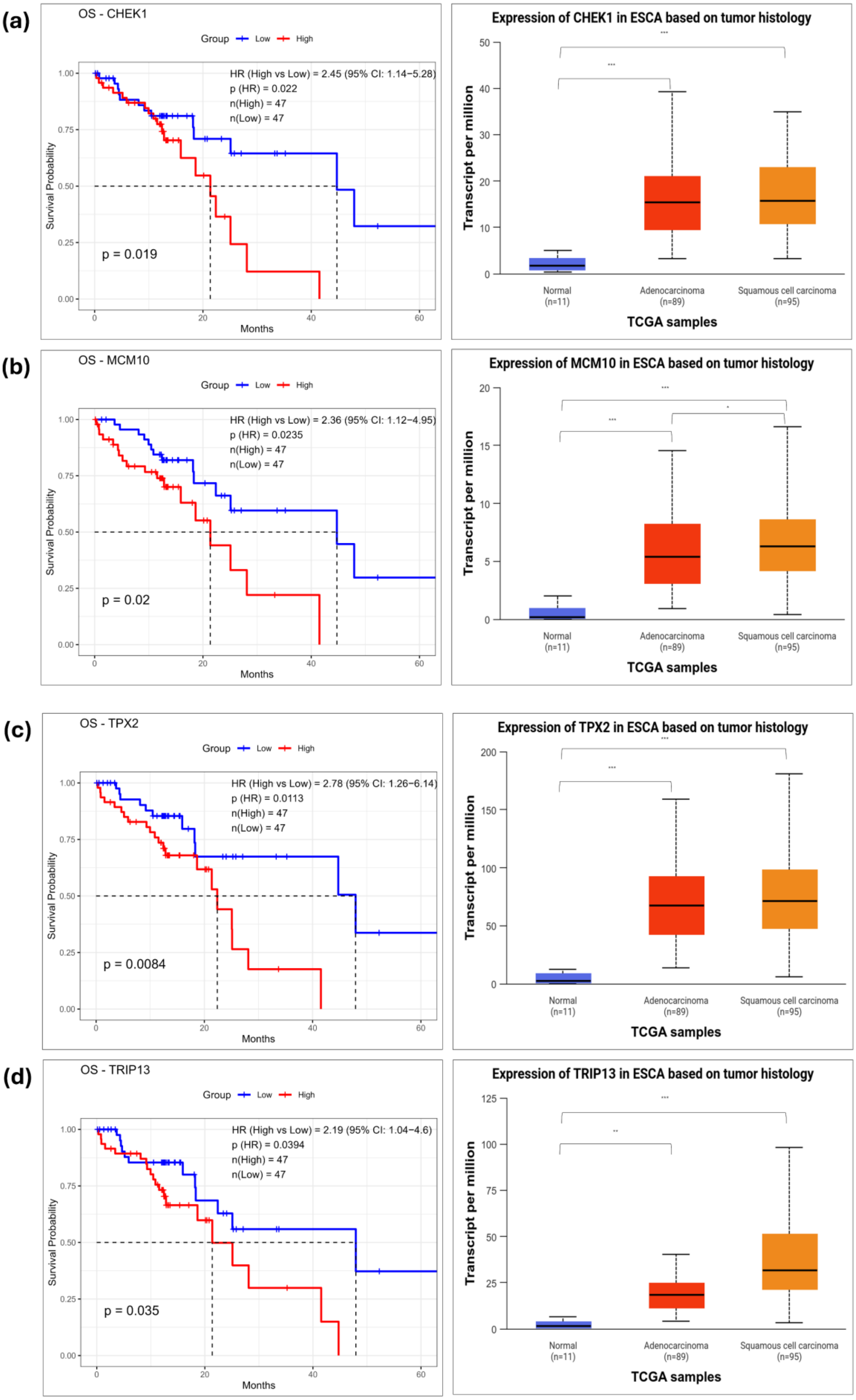

Supplementary Figure S6. Prognostic significance and expression patterns of survival-associated hub genes in the TCGA-ESCA dataset. Kaplan–Meier survival curves (left) and boxplots of mRNA expression (right) for seven hub genes from the DEG-ESCC dataset: High expression of (a) CHEK1, (b) MCM10, (c) TPX2, and (d) TRIP13 was significantly associated with worse overall survival. Statistical significance in the boxplots was annotated as follows: $p < 0.05$ (*), $p < 0.01$ (**), and $p < 0.001$ (***).

(a)

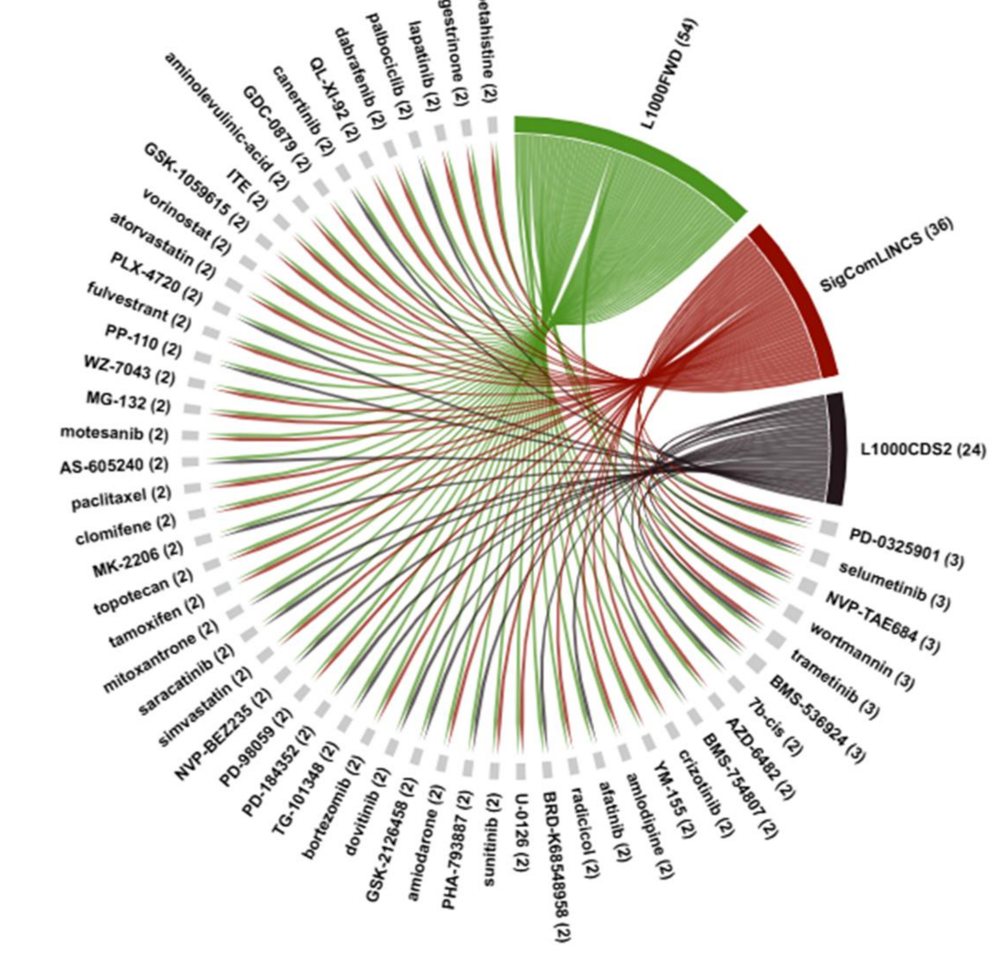
L1000FWD (54)
SigComLINCS (36)
L1000CDS2 (24)
PD-0325901 (3)
selumetinib (3)
NVP-TAE684 (3)
wortmannin (3)
trametinib (3)
BMS-536924 (3)
7b-cis (2)
AZD-6482 (2)
BMS-754807 (2)
crizotinib (2)
YM-155 (2)
amlodipine (2)
afatinib (2)
radicicol (2)
BRD-K68548958 (2)
U-0126 (2)
sunitinib (2)
PHA-793887 (2)
amiodarone (2)
GSK-2126458 (2)
dovitinib (2)
bortezomib (2)
TG-101348 (2)
PD-184352 (2)
PD-98059 (2)
NVP-BEZ235 (2)
simvastatin (2)
saracatinib (2)
mitoxantrone (2)
tamoxifen (2)
topotecan (2)
MK-2206 (2)
clomifene (2)
paclitaxel (2)
AS-605240 (2)
motesanib (2)
MG-132 (2)
WZ-7043 (2)
PP-110 (2)
fulvestrant (2)
PLX-4720 (2)
atorvastatin (2)
vorinostat (2)
GSK-1059615 (2)
ITE (2)
aminolevulinic-acid (2)
GDC-0879 (2)
canertinib (2)
QL-XI-92 (2)
dabrafenib (2)
palbociclib (2)
lapatinib (2)
gestrinone (2)
betahistine (2)

(b)

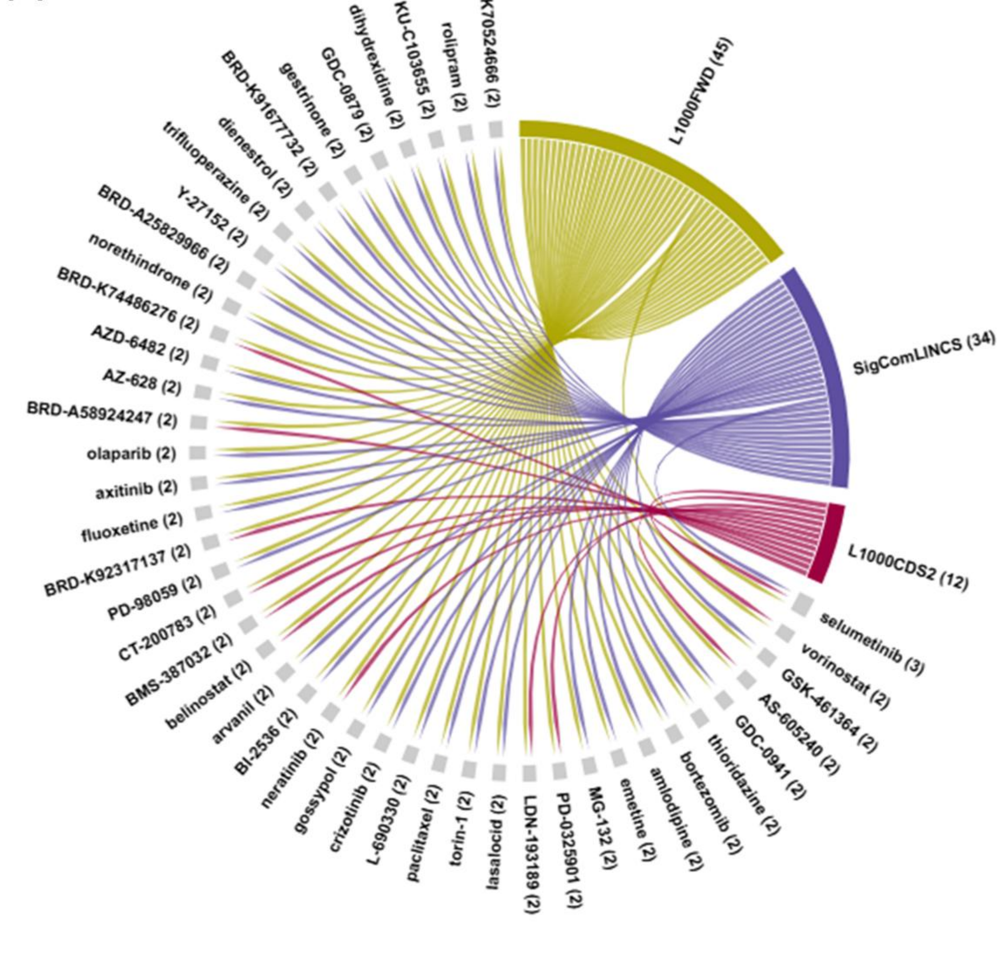
L1000FWD (45)
SigComLINCS (34)
L1000CDS2 (12)
selumetinib (3)
vorinostat (2)
GSK-461364 (2)
AS-605240 (2)
GDC-0941 (2)
thioridazine (2)
bortezomib (2)
amlodipine (2)
emetine (2)
MG-132 (2)
PD-0325901 (2)
LDN-193189 (2)
lasalocid (2)
torin-1 (2)
paclitaxel (2)
L-690330 (2)
crizotinib (2)
gossypol (2)
neratinib (2)
BI-2536 (2)
arvanil (2)
belinostat (2)
BMS-387032 (2)
CT-200783 (2)
PD-98059 (2)
BRD-K92317137 (2)
fluoxetine (2)
axitinib (2)
olaparib (2)
BRD-A58924247 (2)
AZ-628 (2)
AZD-6482 (2)
BRD-K74486276 (2)
norethindrone (2)
BRD-A25829966 (2)
Y-27152 (2)
trifluoperazine (2)
dienestrol (2)
BRD-K91677732 (2)
gestrinone (2)
GDC-0879 (2)
dihydrexidine (2)
KU-C103655 (2)
rolipram (2)
BRD-K70524666 (2)

Supplementary Figure S7. Chord diagrams summarize the potential compounds identified across the three platforms (L1000FWD, L1000CDS2, SigCom LINCS) for each DEG dataset: (a) DEG-ESCC dataset; (b) DEG-EAC&ESCC dataset. Each line connects a platform to its identified compound. In panel (a), black lines indicate compounds identified by L1000CDS2, green lines by L1000FWD, and red lines by SigCom LINCS. In panel (b), red lines indicate compounds identified by L1000CDS2, yellow lines by L1000FWD, and purple lines by SigCom LINCS.
