# Supplementary material for: Evaluating Potential Therapeutic Targets and Drug Repurposing Based on the Esophageal Cancer Subtypes
Source: Pharmaceuticals (Basel). 2025 Aug 11;18(8):1181. doi: 10.3390/ph18081181 (PMC12389467; doi:10.3390/ph18081181)
Supplement: Supplementary file 1 [file pharmaceuticals-18-01181-s001.zip › Supplementary Table S8.pdf]

Supplementary Table S8. Overlapping compounds identified from pairwise comparisons of the three platforms.

DEG-EAC dataset

**Platforms**	**L1000FWD and L1000CDS^2**	**L1000FWD and SigCom LINCS**
Overlapping compounds	mitoxantrone<br>saracatinib<br>CT-200783<br>BI-2536<br>PD-0325901<br>withaferin-a<br>vorinostat<br>selumetinib<br>BRD-K92317137<br>AS-605240<br>trametinib<br>LY-2183240<br>gemcitabine	fluoxetine<br>pioglitazone<br>GDC-0941<br>MG-132<br>doxorubicin<br>TG-101348<br>AZD-6482<br>AZ-628<br>lenalidomide<br>wortmannin<br>ezetimibe<br>econazole<br>NPK76-II-72-1<br>GSK-461364<br>ALW-II-38-3<br>trametinib<br>dasatinib<br>PD-184352<br>exemestane<br>L-690330<br>enzastaurin<br>SB-239063<br>troglitazone<br>rolipram<br>BRD-K67306351<br>norethindrone<br>valdecoxib<br>epothilone<br>SB-525334<br>olaparib<br>BRD-K49553303<br>milnacipran

DEG-ESCC dataset

**Platforms**	**L1000FWD and L1000CDS^2**	**L1000FWD and SigCom LINCS**
Overlapping compounds	7b-cis<br>PD-0325901<br>BMS-754807<br>selumetinib<br>NVP-TAE684<br>wortmannin<br>afatinib<br>BRD-K68548958<br>PHA-793887<br>GSK-2126458<br>dovitinib<br>TG-101348<br>PD-184352<br>trametinib<br>BMS-536924<br>NVP-BEZ235<br>saracatinib<br>mitoxantrone<br>MK-2206	PD-0325901<br>AZD-6482<br>selumetinib<br>NVP-TAE684<br>wortmannin<br>crizotinib<br>YM-155<br>amlodipine<br>radicicol<br>U-0126<br>sunitinib<br>amiodarone<br>bortezomib<br>trametinib<br>BMS-536924<br>PD-98059<br>simvastatin<br>tamoxifen<br>topotecan

	AS-605240<br>PP-110<br>PLX-4720<br>canertinib<br>palbociclib	clomifene<br>paclitaxel<br>motesanib<br>MG-132<br>WZ-7043<br>fulvestrant<br>atorvastatin<br>vorinostat<br>GSK-1059615<br>ITE<br>aminolevulinic-acid<br>GDC-0879<br>QL-XI-92<br>dabrafenib<br>lapatinib<br>gestrinone<br>betahistine

DEG-EAC&ESCC dataset

Platforms	L1000FWD and L1000CDS^2	L1000FWD and SigCom LINCS
Overlapping compounds	vorinostat<br>AS-605240<br>PD-0325901<br>LDN-193189<br>neratinib<br>belinostat<br>BMS-387032<br>CT-200783<br>BRD-K92317137<br>BRD-A58924247<br>selumetinib<br>BRD-K74486276	GSK-461364<br>GDC-0941<br>thioridazine<br>bortezomib<br>amlodipine<br>emetine<br>MG-132<br>lasalocid<br>torin-1<br>paclitaxel<br>L-690330<br>crizotinib<br>gossypol<br>BI-2536<br>arvanil<br>PD-98059<br>fluoxetine<br>axitinib<br>olaparib<br>AZ-628<br>selumetinib<br>AZD-6482<br>norethindrone<br>BRD-A25829966<br>Y-27152<br>trifluoperazine<br>dienestrol<br>BRD-K91677732<br>gestrinone<br>GDC-0879<br>dihydrexidine<br>KU-C103655<br>rolipram<br>BRD-K70524666
